# Apoptosis Induction by OTA and TNF-α in Cultured Primary Rat Hepatocytes and Prevention by Silibinin

**DOI:** 10.3390/toxins4111139

**Published:** 2012-11-02

**Authors:** Ebtisam Essid, Yousef Dernawi, Ernst Petzinger

**Affiliations:** 1 Institute of Pharmacology and Toxicology, Faculty of Veterinary Medicine, Justus-Liebig-University Giessen, Schubert Street 81, Giessen D-35392, Germany; Email: ebtisam_essid@yahoo.de; 2 Institute of Pathology, Faculty of veterinary Medicine, Justus-Liebig-University Giessen, Frankfurter Street 96, Giessen D-35392, Germany; Email: dernawi76@gmail.com

**Keywords:** ochratoxin A, tumor necrosis factor alpha, UV-C light, hydrogen peroxide, rat hepatocytes, DNA ladder, caspase-3, apoptosis, malondialdehyde, silibinin

## Abstract

In cultures of primary rat hepatocytes, apoptosis occurred after application of 20 ng/mL tumor necrosis factor alpha (TNF-α). However, this was only in the presence of 200 ng/mL of the transcriptional inhibitor actinomycin D (ActD). This toxic effect was completely prevented in the presence of 25 µg/mL soluble TNF-α receptor I (sTNFR I) in the supernatant of hepatocyte cell cultures. Apoptosis also occurred after application of 12.5 µmol/L ochratoxin A (OTA). However, that was not prevented by up to 500 µg/mL sTNFR I, indicating that TNF-α/TNFR I is not involved in OTA mediated apoptosis in hepatocytes. The antioxidative flavanolignan silibinin in doses from 130 to 260 µmol/L prevented chromatin condensation, caspase-3 activation, and apoptotic DNA fragmentation that were induced by OTA, by 10 mmol/L hydrogen peroxide (H_2_O_2_) and by ultraviolet (UV-C) light (50 mJ/cm^2^), respectively. To achieve protection by silibinin, the drug was applied to the cell cultures for 2 h in advance. OTA stimulated lipid peroxidation on cultured immortalized rat liver HPCT cells, as was revealed by malondialdehyde (MDA) production. Lipid peroxidation occurred further by H_2_O_2_ and ActD/TNF-α incubation. These reactions were also suppressed by silibinin pretreatment. We conclude that the anti-apoptotic activity of silibinin against OTA, H_2_O_2_ and ActD/ TNF-α is caused *in vitro* by the antioxidative effects of the flavanolignan. Furthermore, cytotoxicity of the pro-apoptotic toxins was revealed by MTT-test. When applied separately, ActD and TNF-α showed no cytotoxic effects after 24 h, but were cytotoxic if applied in combination. The used concentrations of OTA, H_2_O_2_ and the dose of UV-C caused a substantial decrease in viability within 36 h that was prevented mostly by silibinin. We conclude that silibinin is a potent protective compound against apoptosis and cytotoxicity caused by OTA and the investigated compounds.

## 1. Introduction

Liver injury by hepatotoxins may merge in liver cell apoptosis, *i.e.*, the process of programmed cell death. This program permits the removal of damaged cells in multicellular tissue forming-organisms [1]. It results in several morphological changes, e.g., cell shrinkage, membrane blebbing and chromatin condensation along with biochemical events leading to DNA fragmentation and activation of caspases [2,3].

OTA is an unavoidable mycotoxin contaminant of feed and foodstuff that mainly causes nephrotoxicity, as well as hepatotoxicity [4,5]. The hepatotoxic effects of OTA are characterized *in vivo* by apoptotic changes in rat and mouse livers [5,6,7], but also in cultures of isolated rat hepatocytes *in vitro* [8,9]. The apoptotic effects of OTA in liver tissue were thought to be mediated by TNF-α [8] that is released from Kupffer cells [10]. The main apoptotic effects of TNF-α are mediated by its receptor TNFR I [11,12,13,14] and requires the presence of a transcriptional inhibitor *in vitro*, such as ActD [15,16]. However, in tissue cultures of pure isolated rat hepatocytes, OTA mediated apoptosis even in the absence of Kupffer cells and TNF-α [9]. We considered two possible OTA mechanisms leading to apoptosis: (1) direct binding to TNFR I in the absence of TNF-α, thus mimicking cytokine-induced apoptosis; or (2) induction of apoptosis by oxidative stress signals. Therefore, (i) apoptosis by OTA was investigated in the presence of soluble TNFR I in the cell culture medium in order to trap the mycotoxin and (ii) compared with apoptosis mediated by reactive oxygen species (ROS) inducing agents H_2_O_2_ and UV-C light in the absence and presence of the membrane protecting antioxidant silibinin. We show that OTA-mediated apoptosis is likely to be caused by oxidative stress, and that silibinin blocked apoptosis and cytotoxicity. That flavanolignan is extracted from the milk thistle *Silybum marianum* and was used here as a potent antioxidant [17,18,19] and liver cytoprotective agent [20]. Silibinin already blocked the activation of caspase-3 in the HepG2 cell line and in primary rat hepatocytes, providing anti-apoptotic activity [9,21]. 

## 2. Results and Discussion

Liver toxicity is one of the consequences of OTA, but the mechanisms by which OTA exerts its hepatotoxicity are still scantily understood. One possibility is oxidative stress of liver cells by OTA via lipid peroxidation, since the release of ROS under OTA was reported from rat liver [22,23]. Another detrimental cause could be liver cell damage exerted by the release of TNF-α, since OTA provokes TNF-α release in the intact rat liver model from Kupffer cells [20]. However, we showed already that OTA causes apoptosis as a specific cytotoxic event [8], even in the absence of TNF-α [9].

**Figure 1:**
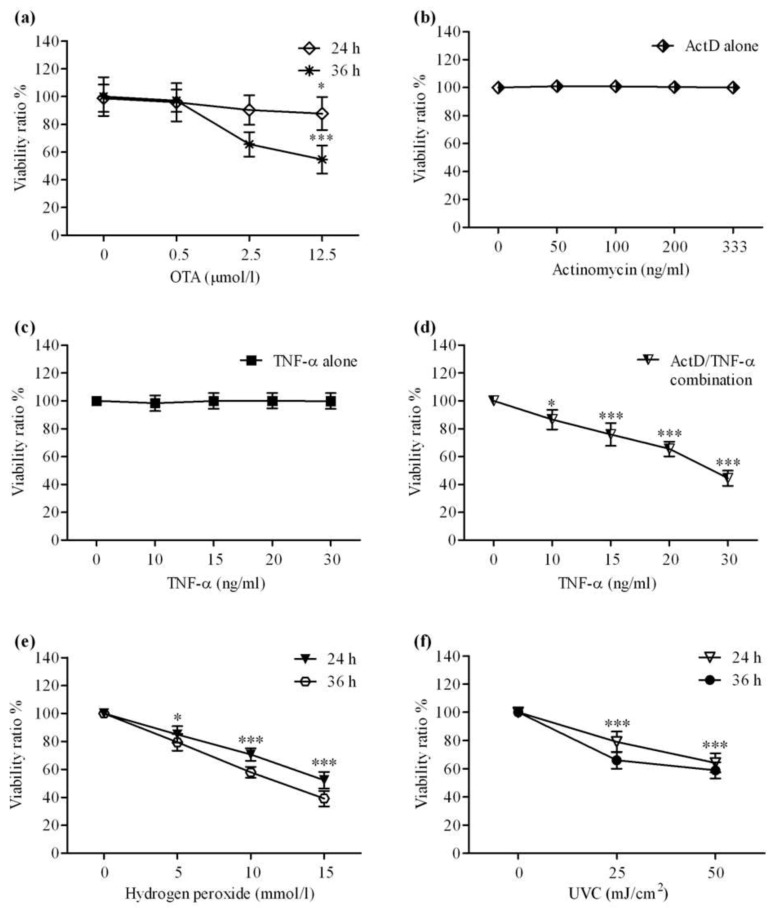
Cytotoxicity of OTA, ActD, TNF-α, H_2_O_2_ and UV-C on cultured primary rat hepatocytes was measured by MTT-test after treatment of primary rat hepatocytes with various concentrations of (**a**) OTA; (**b**) ActD; (**c**) TNF-α alone; (**d**) ActD and TNF-α in combination for 24 h; (**e**) H_2_O_2_; and (**f**) irradiated with UV-C. All experiments were repeated with five different cell preparations. Data presents the value of the mean ± SD (*n* = 5 per group). *p* values * ≤ 0.05, ** ≤ 0.01, *** ≤ 0.001 compared with control values were considered statistically significant.

### 2.1. Cytotoxicity Effects of OTA, ActD, TNF-α, H_2_O, and UV-C

OTA is hepatotoxic in rats, perhaps as a consequence of oxidative stress [23]. It was discussed that OTA reduces the antioxidant defense of cells by down-regulating the expression of several Nrf 2 (nuclear regulator factor 2) and/or HNF4α (hepatic nuclear factor 4α) dependent protective enzymes for antioxidation [24,25], thereby converting cells to be more susceptible to oxidative stress [5].

OTA is cytotoxic on cultured primary rat hepatocytes in a dose- and time-dependent manner (Figure 1a). At 2.5 and 12.5 μmol/L (1 μg/mL and 5 μg/mL), it decreased cell viability after 36 h incubation to 65% and 55%, respectively.

Cell death by TNF-α in cultured hepatocytes becomes manifested only under the metabolic condition of the transcriptional arrest [15]. Therefore, we added ActD separately and in combination with TNF-α to cultured hepatocytes and measured cell toxicity by MTT-test. First, a dose-finding study with ActD in primary rat hepatocyte cultures was performed. Cultures of hepatocytes tolerated 50 to 333 ng/mL of ActD for 24 h without any change in cell viability (Figure 1b). Next, a high and tolerated concentration of ActD (200 ng/mL) was applied for 30 min followed by the addition of increasing concentrations of TNF-α (10–30 ng/mL). The used TNF-α concentrations were tested separately and also were found to be non-toxic (Figure 1c). However, sensitization by ActD caused a vast reduction in cell viability by TNF-α in a dose-dependent manner, leaving 36% of viability at the highest applied TNF-α concentration after 24 h (Figure 1d). These experiments indicated that primary rat hepatocytes are extremely resistant to direct toxic effects of TNF-α, unless pre-sensitized by a transcriptional inhibitor, confirming a previous finding [15]. 

Cytotoxicity is triggered by direct oxidative stress on hepatocytes following the addition of H_2_O_2_ [26] and physically by UV-C radiation, thereby creating reactive oxygen species [27,28]. Here, exposure of primary rat hepatocytes to H_2_O_2_ and UV-C resulted in a dose-and time-dependent cytotoxicity (Figure 1e,f). The cell damage caused decrease of cell viability that was strongest at 15 mmol/L H_2_O_2 _or 50 mJ/cm^2^ UV-C after 36 h.

### 2.2. Effect of Silibinin on OTA, ActD/TNF-α, H_2_O_2_ and UV-C Cytotoxicity

**Figure 2:**
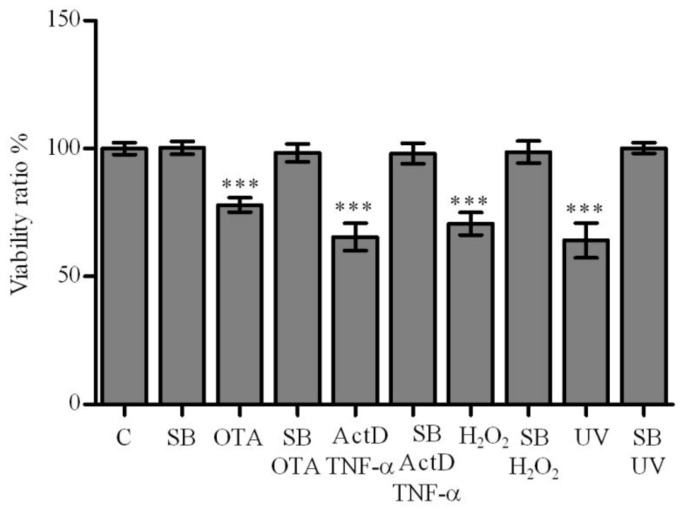
Protective effect of silibinin on ActD/TNF-α, OTA, H_2_O_2_ and UV-C-mediated cytotoxicity in primary rat hepatocyte cultures. Protection was measured by MTT assay after pretreatment of cultured primary rat hepatocytes with silibinin at 260 μmol/L 2 h prior to 200/20 ng/mL ActD/TNF-α and 130 μmol/L 2 h prior to 12.5 μmol/L OTA, 10 mmol/L H_2_O_2_ and 50 mJ/cm^2^ UV-C for 24 h. All experiments were repeated with five different cell preparations. Data presents the value of the mean ± SD (*n* = 5 per group). *p* values: * ≤ 0.05, ** ≤ 0.01, *** ≤ 0.001 compared with control values were considered statistically significant.

Silibinin has been identified as a potent antioxidant with anti-inflammatory, cytoprotective, antifibrotic, anti-lipid peroxidation and anti-carcinogenic effects [29,30,31,32]. Silibinin acts on liver cell membranes to prevent the entry of toxic substances and on the nucleus to accelerate cell regeneration by stimulating protein synthesis [33]. Silibinin was able to restore biochemical alterations caused by hepatotoxicants, such as carbon tetrachloride (CCl_4_) and paracetamol (AAP) through counteracting lipid peroxidation and enzyme leakage [34]. Therefore, we determined the protective effects of silibinin against cytotoxicity in hepatocyte cultures after 24 h incubation with 12.5 µmol/L OTA, with 200/20 ng/mL ActD/TNF-α, with 10 mmol/L H_2_O_2_ or with 50 mJ/cm^2^ UV-C. Under all these conditions, pretreatment for 2 h with silibinin at 130 µmol/L protected the cells as confirmed by MTT-test. Under the same experimental conditions, even the ActD/TNF-α-mediated hepatotoxicity was blocked by silibinin albeit at a higher concentration of 260 µmol/L (Figure 2). 

### 2.3. Effect of OTA, ActD/TNF-α, H_2_O_2_ and UV-C on Caspase-3 Activity and Anti-apoptotic Effects of Silibinin

The activation of caspase-3 represents an early hallmark during apoptosis [35,36,37]. OTA has been shown to induce apoptosis, mainly in kidney and liver cells [5,38]. OTA stimulates physiological pro-apoptotic stimuli such as TNF-α release [10] and reduces anti-apoptotic signaling cascades [39]. Previous studies concerning OTA found this mycotoxin to enhance caspase activities in kidney cells of dogs [40], monkeys [41] and rats [42], as well as in cultured primary rat hepatocytes [8,9]. Moreover, OTA potentiates the effect of TNF-α on the caspase-3-activity in MDCK-C7 cells [40]. 

**Figure 3:**
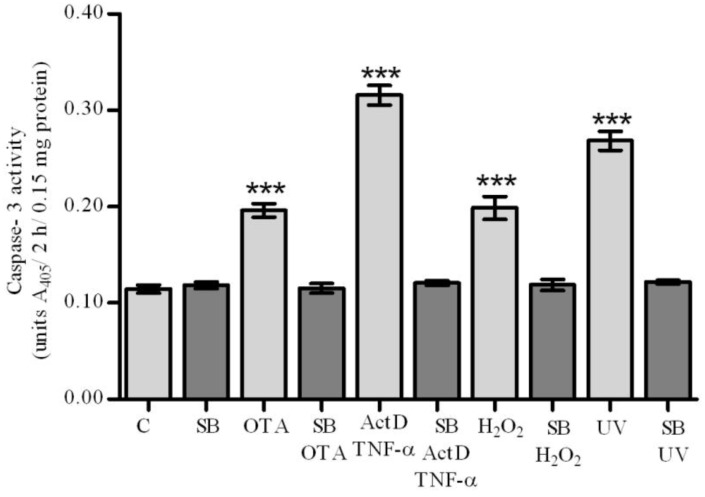
Activation of caspase-3 by OTA, ActD/TNF-α, H_2_O_2_ and UV-C and prevention by silibinin. Caspase-3 activity was measured by a caspase-3/CPP32 colorimetric assay kit after pre-treatment of cultured primary rat hepatocytes with silibinin at 130 μmol/L 2 h prior to 12.5 μmol/L OTA, 10 mmol/L H_2_O_2_ and 50 mJ/cm^2^ UV-C, and 260 μmol/L 2 h prior to 200/20 ng/mL ActD/TNF-α for 12 h. All experiments were repeated with three different cell preparations. Data presents the value of the mean ± SD (*n* = 3 per group). *p* values * ≤ 0.05, ** ≤ 0.01, *** ≤ 0.001 compared with control values were considered statistically significant.

In our case, the exposure of hepatocyte cultures to OTA, ActD/TNF-α, H_2_O_2_ and UV-C, caused activation of caspase-3. The most prominent activation was observed after treatment of primary rat hepatocytes by ActD/TNF-α. Under these conditions, silibinin exerted hepatoprotection (Figure 3). As shown in Figure 3, silibinin completely abrogated OTA-, UV-C- and H_2_O_2_-mediated caspase-3 activation at 130 µmol/L, whereas twice the concentration (260 µmol/L) prevented caspase-3 activation by ActD/TNF-α. Others also found that silibinin inhibits caspases in various cell systems and conditions: it completely protects ECV-304 cells against H_2_O_2_ induced injury mediated by caspase-3 [43], reverses UV-induced HaCaT cell apoptosis by inhibition of caspase-8 after reduction of the expression of FADD (Fas-associated death domain protein) [44], alleviates the activation of caspase-9 and caspase-3 induced by mitomycin C in human melanoma A375-S2 cell [45], prevents the induction of caspase-3 activity in cultured primary rat cardiac myocytes by isoproterenol [46] and in cultured primary rat hepatocytes by tert-butyl hydroperoxide (t-BHP) [47]. 

### 2.4. The Nuclear Damage Caused by OTA, ActD/TNF-α, H_2_O_2_ and UV-C and Prevention by Silibinin

**Figure 4:**
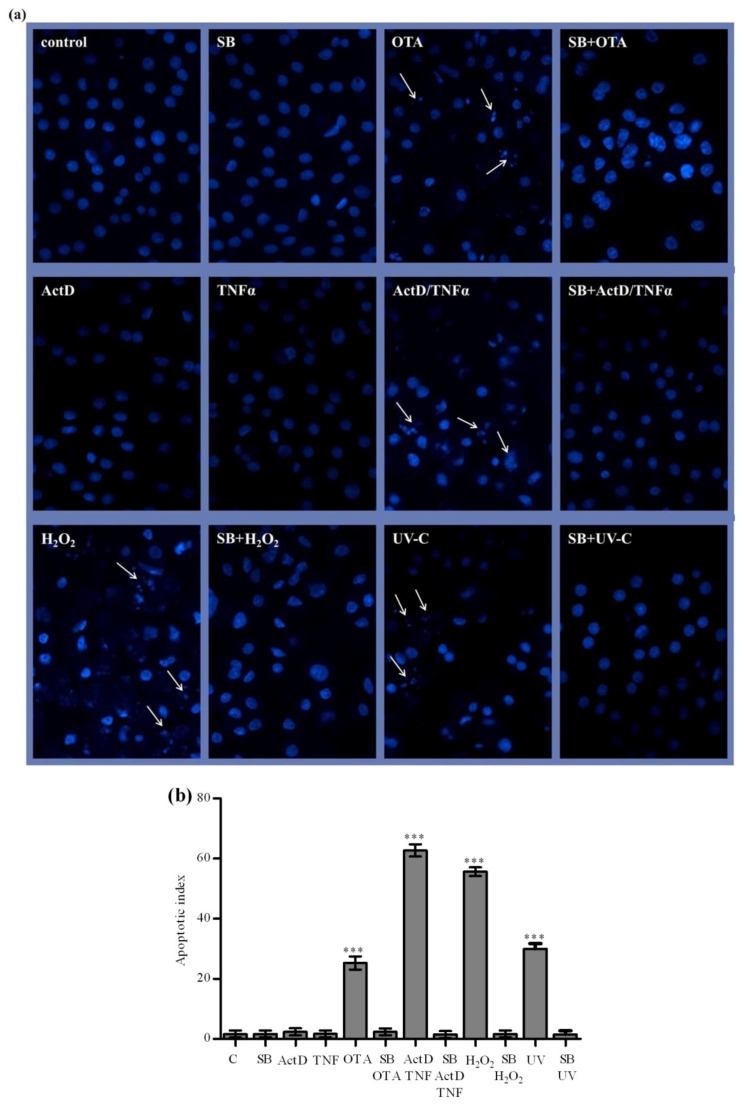
Protective effect of silibinin on OTA, ActD/TNF-α, H_2_O_2_ and UV-C-mediated apoptosis in primary rat hepatocytes was observed after pretreatment with 260 µmol/L silibinin given 2 h prior to 12.5 µmol/L OTA, 200/20 ng/mL ActD/TNF-α, 10 mmol/L H_2_O_2_ and 50 mJ/cm^2^ UV-C for 24 h. Cultured cells were fixed, chromatin was stained with Hoechst, and apoptotic nuclei were counted. (**a**) Fluorescence micrographs of Hoechst stained nuclei of primary rat hepatocytes (×400). The white arrows indicate apoptotic nuclei exhibiting fragmented chromatin, whereas other nuclei are intact. The pictures are representing at least five fields from a slide; each experiment was performed at least 3 times; (**b**) The percentage of apoptotic nuclei is given as apoptotic index. *n* = 3, treatment groups were compared to control using one-way ANOVA. *p* values * ≤ 0.05, ** ≤ 0.01, *** ≤ 0.001 compared with control values were considered statistically significant.

In order to document the extent of nuclear damage under apoptosis, we stained the chromatin with Hoechst 33258 and counted apoptotic nuclei using fluorescence microscopy. Figure 4a shows micrographs of apoptotic nuclei (white arrows) in cultured primary rat hepatocytes exposed to OTA, ActD/TNF-α, H_2_O_2_ and UV-C. Treatment of primary rat hepatocytes by OTA, ActD/TNF-α, H_2_O_2_ and UV-C led to a statistically significant induction rate of nuclear apoptotic events on approximately 25, 62, 55 and 30% of cells, respectively (Figure 4b). These correlate with cytotoxicity as examined with MTT-test (Figure 1).

### 2.5. Development of DNA Ladders by OTA, ActD/TNF-α, H_2_O_2_ and UV-C and Prevention by Silibinin

**Figure 5:**
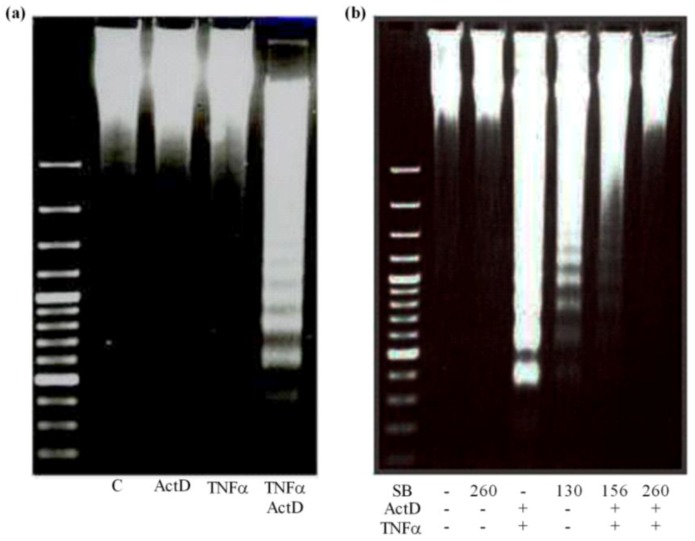
Development of DNA ladder by ActD/TNF-α and prevention by silibinin. Cultures of primary rat hepatocytes were treated (**a**) with ActD 200 ng/mL, TNF-α 20 ng/mL, and ActD 200 ng/mL 30 min prior to 20 ng/mL TNF-α; the first three lanes show high-molecular, non-fragmented DNA; or (**b**) pre-treated with silibinin at increasing concentrations 2 h prior to 200/20 ng/mL ActD/TNF-α. Then, after 24 h the DNA was isolated and visualized by gel electrophoresis. All experiments were repeated with five different cell preparations.

The late stage of apoptosis is characterized by activation of endonucleases that cleave chromosomal DNA leading to the formation of mono- and oligonucleosomal DNA fragments with a length of approximately 180–200 bps and multiples showing characteristic ladder pattern on agarose gel. No fragmentation was observed in control cultures. Neither TNF-α nor ActD alone developed DNA fragmentation in cultured primary rat hepatocytes. By contrast, the combination of ActD with TNF-α generated DNA ladders after 24 h (Figure 5a). Silibinin dose-dependently decreased DNA fragmentation and even completely abolished fragments at high concentration after 24 h (Figure 5b). 

**Figure 6:**
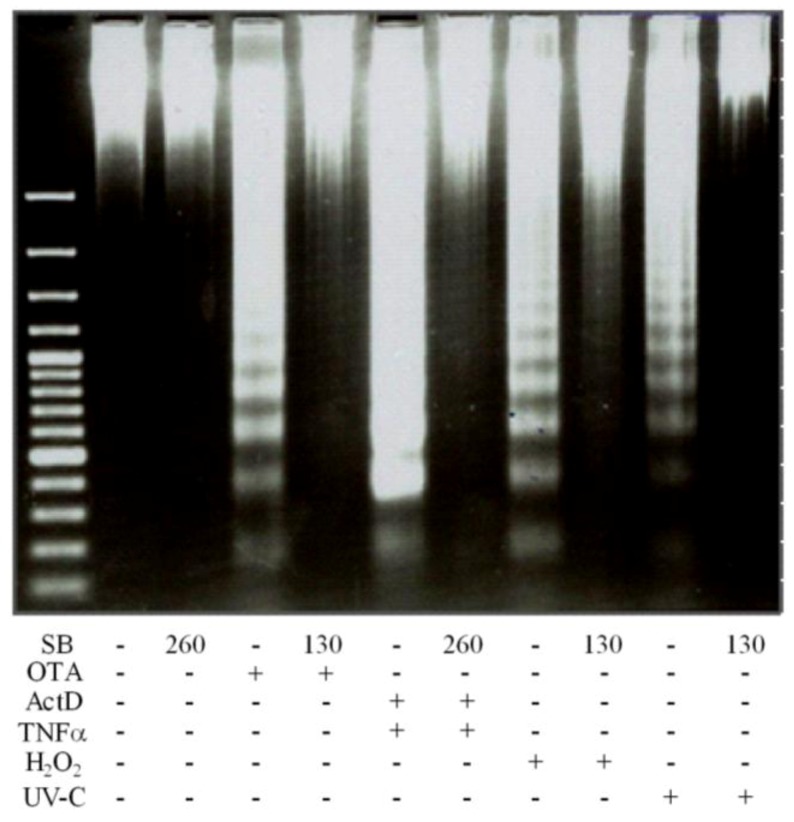
Development of DNA ladder by OTA, ActD/TNF-α, H_2_O_2_ and UV-C and prevention by silibinin. Primary rat hepatocytes were pretreated with silibinin at 130 µmol/L 2 h prior to 12.5 µmol/L OTA, 10 mmol/L H_2_O_2_ and 50000 µJ/cm^2^ and at 260 µmol/L 2 h prior to 200/20 ng/mL ActD/TNF-α. Then, after 24 h, the DNA was isolated and visualized by gel electrophoresis. All experiments were repeated with five different cell preparations.

*In vitro* and *in vivo* studies described that silibinin shields the liver from oxidative stress and persistent inflammatory processes, mainly caused by reactive oxygen species and pro-inflammatory cytokines [32]. It also reduced the signs of oxidative stress in hepatocytes and elevated mitochondrial ATP levels [48]. Therefore, we tested its preventive effect on OTA, H_2_O_2_ and UV-C mediated DNA ladder formation. Remarkably, the findings indicated that silibinin pretreatment completely prevented DNA laddering induced by OTA, ActD/TNF-α, H_2_O_2_ and UV-C (Figure 6). In conclusion, we consider silibinin as a quite strong anti-apoptotic compound that exerts protection against OTA, ActD/TNF-α, H_2_O_2_ and UV-C mediated apoptosis, most likely by its antioxidant and membrane stabilizing effect on liver cells [19,32].

### 2.6. OTA and Soluble TNF-α Receptor I (sTNFRI)

**Figure 7:**
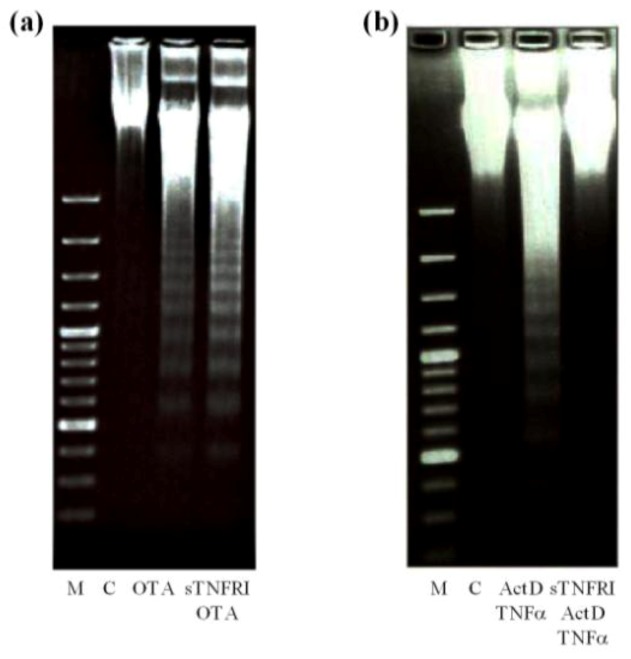
Effect of sTNFRI on OTA and ActD/TNF-α-mediated DNA fragmentation. Primary rat hepatocytes were pre-treated with sTNFRI at 500 µg/mL 2 h prior to 12.5 µmol/L OTA (**a**); and at 25 µg/mL 2 h prior to 200/20 ng/mL ActD/TNF-α (**b**). Then, after 24 h, the DNA was isolated and visualized by gel electrophoresis. All experiments were repeated with five different cell preparations.

The generally accepted picture of receptor-mediated apoptosis by TNF-α claims that the cell membrane receptor TNF-α receptor I (TNFRI) triggers apoptosis by binding TNF-α with its extracellular domain and by activation of its intracellular domain, called adaptor protein TNFRI associated death domain (TRADD). Following activation of TRADD, the recruitment and activation of the initiator aspartate specific cysteine protease caspase-8 occurs, which triggers further events of receptor induced apoptosis [49,50]. In order to gain insight into the mechanism of apoptosis by OTA, primary rat hepatocyte cultures were pre-incubated with soluble sTNFRI as a decoy strategy to reduce the binding of OTA to the membrane receptor by competition and to decrease the potency for OTA-mediated apoptosis. Figure 7a shows that addition of 500 µg/ mL sTNFRI 2 h prior to 12.5 µmol/L OTA had no effect on OTA-mediated DNA laddering. However, only 25 µg/ mL sTNFRI 2 h prior to 200/20 ng/mL ActD/TNF-α completely prevented TNF-α-mediated DNA fragmentation (Figure 7b) Therefore, a direct activation of the TNF-receptor I by OTA is unlikely. 

**Figure 8:**
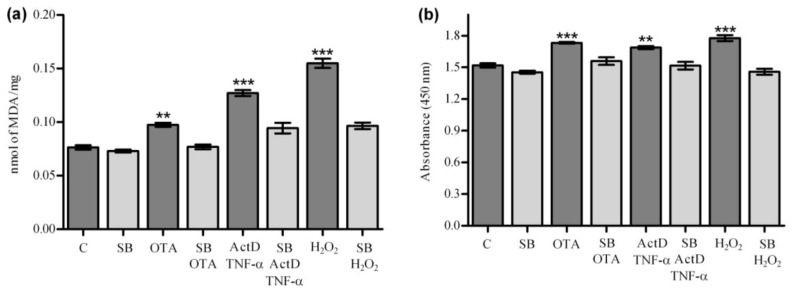
Silibinin effect on oxidative stress induced by OTA, ActD/TNF-α and H_2_O_2_. HPCT-1E3 cells were pre-treated with silibinin at 260 µmol/L 2 h prior to 50 µmol/L OTA, 200/20 ng/mL ActD/TNF-α and 10 mmol/L H_2_O_2_. Then, after 6 h, the lipid peroxidation was determined fluorometrically (**a**) and the ROS generation was measured colorimetrically (**b**). All experiments were repeated with 3 different cell cultures. *p* values * ≤ 0.05, ** ≤ 0.01, *** ≤ 0.001 compared with control values were considered statistically significant.

### 2.7. OTA Causes Cell Toxicity *In Vitro* via Oxidative Stress Reactions

Our results so far point out that, alternatively to cytokine mediated apoptosis and cytotoxicity, oxidative stress reactions may have caused cell damage and that silibinin has prevented them as an antioxidant. A test was performed to detect reactive oxygen species and also malondialdehyde as a natural end product of membrane lipid peroxidation. Cell cultures of immortalized rat hepatocytoma cells (HPCT) [51,52] were exposed to OTA and ActD/TNF-α. For control, samples were also analyzed after incubation with H_2_O_2_. The results indicated that OTA generates, as with the other hepatotoxins, oxygen radicals in cell cultures (Figure 8).

## 3. Materials and Methods

### 3.1. Chemicals and Reagents

Silibinin (*MW* 482.44 g/mol), Recombinant Rat Tumor Necrosis Factor-α (TNF-α), fetal bovine serum, dexamethasone, inosine, trypan blue and Hoechst stain 33342 were purchased from Sigma Aldrich, Steinheim, Germany. Penicillin, streptomycin, trypsin/EDTA, sodium pyruvate and Dulbeccos modified Eagles medium (DMEM) low glucose were purchased from PAA Laboratories, Cölbe, Germany. Ochratoxin A (*MW* 403.81 g/mol) was purchased from CSIR Food Science and Technology, Pretoria, South Africa. Hydrogen peroxide (H_2_O_2_), DMSO, agarose NEEO Ultra-Quality, and MTT (3-(4,5-dimethylthiazol-2-yl)-2,5-diphenyl tetrazolium bromide) were purchased from Roth, Karlsruhe, Germany. HEPES (*N*-2-hydroxyethylpiperazine-*N*'-2-ethanesulfonic acid) and collagen R and recombinant human insulin were purchased from SERVA Electrophoresis GmbH, Heidelberg, Germany. Proteinase K, RNase and DNA ladder markers were purchased from Fermentas, St. Leon-Rot, Germany. Soluble tumor necrosis factor receptor I (sTNFRI) was kindly obtained from Prof. Dr. Joachim Roth, Giessen.

### 3.2. Animals

Primary rat hepatocytes were isolated from male Wistar rats (200–280 g). The animals were fed *ad libitum* with Altromin^®^ standard diet and received water *ad libitum*. They were kept under 12 h light-dark cycles at 22 °C temperature and ventilation. The health of the rats was routinely tested by sentinel animals, and the animals were found to be free of chronic infections and parasites.

### 3.3. Isolation and Culture of Primary Rat Hepatocytes

Male Wistar rats were anesthetized with urethane 20% (7.5 mg/kg; i.p.). Hepatocytes were prepared by EDTA *in situ* perfusion method as described in [9]. The cell pellets were re-suspended in DMEM low glucose containing 10% fetal calf serum, 1% penicillin and streptomycin. Cell viability was revealed by trypan blue exclusion test and always exceeded 98%. The isolated hepatocytes were seeded on collagen-coated Petri dishes or collagen-coated multiwell plates and left for 3 h to attach at 37 °C in a humidified atmosphere of 5% CO_2_ in air. The culture medium was replaced, and the compounds were applied. Control cells were treated with equal volume of DMSO as a vehicle for OTA and silibinin. Final DMSO concentration was 0.5% for all treatments.

### 3.4. Sensitization of Primary Rat Hepatocytes

After replacement of the culture medium, the isolated, adherent rat hepatocytes were pre-incubated with ActD (200 ng/mL) for 30 min, and then rat recombinant TNF-α was added to some dishes at a concentration of 20 ng/mL, unless otherwise indicated.

### 3.5. Culture of HPCT-1E3 Hepatocytoma Cells

Because HPCT-1E3 cells are a good model for studying cytotoxicity and express several hepatocyte specific properties in contrast to other immortal cell lines, their suitability as an *in vitro* model has to be proven in order to replace *in vivo* experiments [51]. The rat hepatocytoma cell line HPCT-1E3 was maintained in Dulbecco’s modified Eagle’s medium (DMEM) high glucose supplemented with 10% (*v*/*v*) fetal calf serum, 2 mM L-glutamine, 10 µg/mL insulin, 10 µg/mL inosine, 1.5 µmol/L dexamethasone, 100 IU/mL penicillin and 100 mg/mL streptomycin [53].

### 3.6. Cytotoxicity Assay

The concentration dependency of cytotoxicity of ActD, TNF-α, H_2_O_2 _and UV was determined by the 3-(4,5-dimethylthiazol-2-yl)-2,5-diphenyl tetrazolium bromide (MTT) assay [54], detecting the cellular mitochondrial activity to convert MTT tetrazolium salt to water-insoluble formazan. Rat hepatocytes at density of 5 × 10^4^ cells/0.1 mL DMEM were seeded in 96-well collagen-coated tissue culture plates. After 3 h incubation, the cells were treated with different concentrations of either ActD at 0, 50, 100, 200 and 333 ng/mL, or with TNF-α at 0, 10, 15, 20 and 30 ng/mL. Other cells were pre-incubated with 200 ng/mL ActD 30 min, prior to TNF-α, at the given concentrations for 24 h; others were treated with 0, 0.5, 2.5, 12.5 µmol/L OTA, 0, 5, 10 and 15 mmol/L H_2_O_2_ or irradiated with UV light (254 nm; Stratalinker^®^ UV crosslinker 1800, Stratagene, La Jolla, CA, United States) at 0, 25 and 50 mJ/cm^2^, and all were incubated for 24 and 36 h. To assess the effect of silibinin on the cytotoxicity, cells were pre-treated with and without silibinin at the given concentrations 2 h prior to the administration of ActD/ TNF-α at 200/20 ng/mL or 12.5 µmol/L OTA or 10 mmol/L H_2_O_2, _or irradiation with UV light at 50 mJ/cm^2^. After that, the cells were incubated with 10 µL of MTT solution (5 mg/mL) for 3 h. The formation of formazan crystals were tracked occasionally under the microscope. Subsequently, the medium was aspirated and the formazan crystals were solubilized by adding 100 µL isopropanol solution (98.86 mL 2-propanol and 1.14 mL HCl) and mixed to ensure the dissolving of crystals. Finally, the absorbance of each well was measured by a Benchmark microplate reader at dual wavelength (550 nm and 655 nm). Within 1 h of adding the isopropanol, plates were read. Cell viability was expressed as percentage of formazan absorbance of non-treated viable cells under each treatment regimen.

### 3.7. Analysis of Hepatocyte Nuclear Morphology

In order to visually assess apoptosis induction by OTA, ActD/TNF-α, H_2_O_2_ and UV, we stained the chromatin of fixed primary hepatocyte monolayers with DNA binding fluorochrome Hoechst stain 33342 and counted apoptotic nuclei using fluorescence microscopy. Briefly, 1 mL cell suspension containing 2 × 10^5^ cells were seeded on collagen-coated 2 well Lab-tek tissue culture chamber/slides. After 3 h of incubation at 37 °C, the cells were pre-treated with and without silibinin at the given concentrations 2 h prior to the administration of 12.5 µmol/L OTA, ActD/TNF-α 200/20 ng/mL separately and in combination, 10 mmol/L H_2_O_2_ or irradiated with UV light (254 nm; Stratalinker^®^ UV crosslinker 1800, Stratagene, La Jolla, CA, United States) at a dose of 50 mJ/cm^2^ for 24 h. After that, the cells were washed with cold PBS once and fixed with 2% paraformaldehyde for 10 min. Then, Hoechst 33342 solution was added at 1 µg/mL for 5 min at room temperature in darkness. Finally, stained cells were washed with PBS again, mounted and visualized under a fluorescence microscope. Fluorescence imaging was performed on Nikon Eclipse 80i fluorescence microscope supplied with DAPI filter (excitation 360 nm, emission 460 nm) from Japan. The images were captured with the NIS elements software (V 3.10). Finally, the apoptotic nuclei were counted using Adobe Photoshop CS5 extended version 12.1. The percentage of apoptotic nuclei is given as apoptotic index.

### 3.8. Caspase-3 Activity

Caspase-3 activity was determined using Caspase-3/CPP32 Activity Colorimetric Assay Kit according to the company instructions (PromoCell, Heidelberg, Germany). The assay is based on the spectrophotometric detection of the chromophore p-nitroanilide (pNA) after cleavage from the labeled substrate DEVD-pNA. Briefly, isolated primary rat hepatocytes were seeded in 94 mm Petri dishes and incubated for 3 h. Then, the cells were pre-treated with and without silibinin at 130 µmol/L 2 h prior to the administration of 12.5 µmol/L OTA or 10 mmol/L H_2_O_2_ or irradiation with UV light at a dose of 50 mJ/cm^2^ and at 260 µmol/L 2 h prior to ActD/TNF-α 200/20 ng/mL for 12 h. The cells were harvested after 12 h without trypsinization by a cell scraper from the incubation medium and centrifuged at 420× *g* for 5 min at 25°C. The final pellet was re-suspended in 50 µL of chilled Cell Lysis Buffer (PromoCell) and incubated on ice for 10 minutes followed by centrifugation at 13,000× *g* for 1 min. The supernatant (cytosolic extract) was transferred to a fresh tube and immediately stored at −80 °C. The protein content of the supernatant was reliably estimated with BCA protein assay kit (Novagen). For colorimetric measurement of caspase-3 activity, 150 µg protein of the cytosolic extract was diluted to 50 µL Cell Lysis Buffer for each sample. Subsequently, 50 µL of 2× Reaction Buffer containing 10 mmol/L DTT and 5 µL of the 4 mmol/L DEVD-pNA substrate was added and then incubated at 37 °C for 2 h. The enzyme activity (color intensity from DEVD-pNA cleavage) was measured at 405 nm with a microplate reader.

### 3.9. DNA Ladder Fragmentation Analysis

Isolated primary rat hepatocytes were seeded in 94 mm Petri dishes and incubated for 3 h. Then the cells were pre-treated with and without silibinin at 130 µmol/L 2 h prior to the administration of OTA, H_2_O_2_ or irradiation with UV light or at 260 µmol/L 2 h prior to ActD/TNF-α for 24 h. Other cells were pre-treated with sTNFRI 2 h prior to the administration of OTA and ActD/TNF-α. Cells were harvested after 24 h as described above in the incubation medium and were spun down at 500× *g* for 5 min at 25 °C, re-suspended in PBS and spun down again. Genomic DNA was isolated according to Wörner and Schrenk [55]. DNA concentration was measured at 260 nm using a spectrophotometer. Three micrograms of DNA was resolved on 1.5% agarose gel in TAE buffer (40 mmol/L Tris-HCl, 1 mmol/L EDTA, 20 mmol/L acetic acid, pH 8.0). The gel was stained with ethidium bromide solution 10 mg/ mL and de-stained in water. The DNA bands were visualized under UV illumination and photographed using a gel-video documentation system (Image Master VDS, Pharmacia Biotech, Freiburg, Germany). 

### 3.10. Oxidative Stress Determination

The oxidative stress was assessed: (1) fluorometrically by measuring malondialdehyde (MDA) as an end product of lipid peroxidation using Abcam’s Lipid Peroxidation Assay Kit (Abcam, Cat.-No. ab118970, Cambridge, UK) and (2) colorimetrically by determining ROS generation using ROS ELISA kit (antikörper-online.de, Cat.-No. E02R0069, BlueGene, Shanghai, China). 

HPCT-1E3 cells were seeded at (1 × 10^6^ cells) on 35 mm Petri dishes and pre-incubated overnight. Then, the cells were pre-treated with and without silibinin at 260 µmol/L 2 h prior to the administration of 50 µmol/L OTA, ActD/TNF-α 200/20 ng/mL and 10 mmol/L H_2_O_2_ for 6 h. The cells were harvested after 6 h without trypsinization by a cell scraper from the incubation medium and centrifuged at 3000 rpm for 3 min at 25 °C. The final pellet was used for measuring MDA and ROS according to the company’s instructions.

### 3.11. Statistical Analysis

Hepatocytes were obtained from three to five different animals as indicated by “n”. Experiments were performed with each cell preparation three times in case of caspase assay, eight times in case of MTT test, once in case of DNA laddering and three times in case of oxidative stress. The results were expressed as mean ± SD. Statistical analysis was done using one-way ANOVA followed by Tukey’s multiple comparison test with Graphpad Prism 5.03 software (San Diego, CA, USA).

## 4. Conclusions

The mycotoxin ochratoxin A caused *in vitro* cytotoxicity and apoptosis in rat hepatocyte cell cultures that were independent on TNF-α and were, thus, different from *in vivo* conditions in the intact rat liver. OTA lacked binding to soluble TNF-α receptor I (sTNFRI). In cell cultures, toxicity of OTA and ActD/TNF-α was conveyed by oxidative stress and was comparable to H_2_O_2_. The herbal flavanolignan silibinin was a potent cytoprotective and anti-apoptotic agent that counteracted the cytotoxicity and induction of apoptosis by OTA, ActD/TNF-α, H_2_O_2_ and UV-C on primary rat hepatocytes cultures. The powerful hepatoprotective effects of silibinin were observed in a dose-dependent manner when the compound was added in advance. This distinguishes silibinin as a prophylactic hepatoprotective remedy. 
